# Improvement in genetic evaluation of quantitative traits in sheep by enriching genetic model with dominance effects

**DOI:** 10.1038/s41598-025-16005-5

**Published:** 2025-08-17

**Authors:** Farhad Ghafouri-Kesbi, Morteza Mokhtari, Mohsen Gholizadeh

**Affiliations:** 1 Department of Animal Science, Faculty of Agriculture, Bu-Ali Sina University, Hamedan, Iran; 2 Department of Animal Science, Faculty of Agriculture, University of Jiroft, Jiroft, Iran; 3 Department of Animal Science, Faculty of Animal and Aquatic Science, Sari Agricultural Sciences and Natural Resources University, Sari, Iran

**Keywords:** Sheep, Animal model, Dominance effects, Heritability, Breeding values, Agricultural genetics, Animal breeding

## Abstract

Although dominance effects play a major role in quantitative genetics, most studies on quantitative traits have often neglected dominance effects, assuming alleles act additively. Therefore, the aim followed here was to quantify the proportion of variation in the early growth of Baluchi sheep that was attributed to dominance effects. Data collected over a 28-year period at the Baluchi sheep breeding station was used in this study. Traits evaluated were birth weight (**BW**), weaning weight (**WW**) and average daily gain (**ADG**). Each trait was analyzed with a series of twelve animal models which included different combinations of additive genetic, dominance genetic, maternal genetic and maternal permanent environmental effects. The Akaike’s information criterion (**AIC**) was used to rank models. The predictive ability of models was measured using the mean squared error of prediction (MSE) and Pearson’s correlation coefficient between the real and predicted values of records (r($\:y$,$\:\widehat{y}$)). Correlations between traits due to additive and dominance effects were estimated using bivariate analyses. For all traits studied, including dominance effects improved the likelihood of the fitting model. In addition, models that included dominance effects had the better predictive ability as provided higher r($\:y$,$\:\widehat{y}$) and lower MSE. However, accounting for dominance effects significantly increased the computing burden evidenced by considerably longer computing time and a huge amount of memory required. By including dominance effects in the model, additive genetic variance did not change, but residual variance decreased significantly up to 41%, which indicated that the dominance component distangelled from residual variance. For **BW**, **WW** and **ADG**, dominance genetic variance was 6.61, 1.91, and 2.73 times greater than additive genetic variance and contributed 87%, 65% and 73% to the total genetic variance, respectively. Estimates of dominance heritability ($\:{\varvec{h}}_{\varvec{d}}^{2}$), were 0.29 ± 0.06, 0.15 ± 0.07 and 0.20 ± 0.07 for **BW**, **WW** and **ADG**, respectively. Additive heritability ($\:{\varvec{h}}_{\varvec{a}}^{2}$), was 0.05 ± 0.01 for **BW**, 0.08 ± 0.02 for **WW** and 0.07 ± 0.02 for **ADG**, respectively. By including dominance effects in the model, the accuracy of additive breeding values increased by 8%, 8% and 11% for **BW**, **WW** and **ADG**, respectively. Correlation between additive breeding values obtained from the best model and the best model without dominance effects were close to unity for all traits studied, indicating negligible changes in the additive breeding values and little chance for re-ranking of top animals across models. While additive genetic correlations were all positive and high, the dominance genetic correlation between **WW** and **ADG** was positively high (0.99), and between other pairs of traits was negative. Although the inclusion of dominance effects in the model did not change the ranking of top animals and had high computational requirements, it improved the predictive performance of the model and led to a significantly better data fit and an increase in the accuracy of additive breeding values. Therefore, including dominance effects in the model for genetic evaluation of the early growth of Baluchi lambs can be a reasonable recommendation.

## Introduction

Production traits in farm animals are mostly quantitative traits. They have continuously distributed phenotypes that do not show simple Mendelian inheritance. These traits are controlled by a large number of genes, each of which has a partial effect, and the cumulative and individual effects of such genes, along with the environmental effects, cause continuously distributed phenotypes​​ in the population^1,2^. The phenotypic variation of quantitative traits observed within a population may be due to genetic variation, environmental variation or a combination of both^3^. Animal breeders want to quantify the former as it is necessary for response to selection. The presence of genetic variation in the population shows that there is variation in the genetic merit or so-called “breeding values” of animals for a particular trait. Breeding value, the heritable portion of an individual’s observed phenotypic value, defines the superiority or inferiority of the offspring of an animal and is exploited by breeders to rank and select superior animals to be the parents of the next generation to achieve genetic improvement. Remarkably, this selective breeding, or artificial selection, has a long and successful application in animal breeding, resulting in a significant improvement in the production and reproduction performance of livestock^4^.

Selection on a heritable trait generates a response in the phenotypic distribution of the trait, the extent of which is determined by the magnitude and form of selection and the heritability of the trait^3^. Therefore, to design an effective selection program, information regarding trait heritability and its genetic relationship with other traits is required. By estimating the amount of additive genetic variation in the traits, and genetic correlations between them, we can make predictions about their response to selection^5^.

Many studies have been conducted to estimate the heritability of economic traits in animal and plant species. However, models used in these studies only include additive genetic component^6–11^, and little effort has been made to expand these models to predict genetic merits to account for dominance effects^12^. This is because of some reasons. First, since dominance variance does not contribute to predicting the response to selection, it has not been considered important^13^. The second reason is the cumbersome and complex experimental designs needed for the decomposition of the genetic variance to its additive and dominance components^14^. The third reason is the over-parameterization of the models following the inclusion of dominance effects^15^. The fourth reason is that accounting for dominance effects suffers a large computational burden due to the much more complex computation for constructing the dominance genetic relationship matrix and its inverse which is needed for estimating the dominance genetic component^16^. The last reason is that the estimation of dominance genetic variance is highly sensitive to data structure (i.e., incomplete pedigree) compared to additive genetic variance^15^.

Dominance genetics describes the relationship between the phenotype and the genotype at a diploid locus in heterozygotes. In quantitative genetics, dominance is the phenomenon where the genotypic value of the heterozygote deviates from the mean genotypic value of the two homozygotes^5^. Dominance is not inherent to an allele or its traits (phenotype). It is a strictly relative effect between two alleles of a given gene of any function; one allele can be dominant over a second allele of the same gene, recessive to a third, and co-dominant with a fourth^17^. Although, using QTL mapping approaches, early research has characterized the contribution of dominance effects to the genetic architecture underlying phenotypic variation in murine body weight^18,^^19^, fertility and production traits in cattle^20^ and fatness in chicken^21^, using models including dominance effects for genetic evaluation of production traits in livestock is traced back to the recent decade. Varona et al.^22^ reported that including the dominance effects in the genetic model can (1) lead to a better understanding of the genetic architecture of traits, (2) increase the accuracy of genetic evaluation, and (3) improve the efficiency of breeding programs via determining the relative importance of dominance effects. Garel et al.^14^ reports stated that ignoring dominance effects distorts the prediction of crossover values, as well as genetic variance components and the genetic merit of genotypes. In the presence of dominance, the additive + dominance model may yield more accurate estimates of the average effect of a single gene or allele substitution effect than the purely additive model and, therefore, more accurate estimates of heritability and additive breeding values^23^. Accordingly, in recent years, mixed animal models incorporating restricted maximum likelihood (REML) procedure have been applied to estimate both additive and dominance variances for production traits in goat^15,24^ and chicken^25^, though, to our knowledge, in the framework of animal models (using phenotypic records and pedigree information), the contribution of dominance effects to the phenotypic variation of economic traits in sheep has not been quantified so far.

The Baluchi sheep is a fat-tailed breed well adapted to a wide range of harsh environmental conditions in eastern Iran, one of the arid subtropical areas of the world. This breed comprises 12% of the total sheep population in Iran^26^. The animals of this breed are known for their small size, fat tails, carpet wool and white color with black marks on the legs and head. Their face is generally black. They are generally polled in both genders. Although genetic parameters for growth traits of Baluchi sheep have been estimated by Yazdi et al.^27^, Jalil-Sarghale et al.^28^, Bahreini-Behzadi et al.^26^ and Bahri Binabaj et al.^29^, genetic parameters for dominance effects are not available for this breed. Moreover, there is no previous information regarding the correlation between growth traits caused by dominance effects. Therefore, this study was conducted to estimate dominance effects on the pre-weaning growth traits of Baluchi sheep. The correlation between traits due to dominance effects was also estimated.

## Materials and methods

### Flock and management

The data used in the present study was obtained from the Baluchi sheep breeding station (flock 1) which is located in Mashhad, Khorasan Razavi, Iran. Pedigree and body weight records were available from 1980. This experimental population of Baluchi sheep was founded in the early 1960s. In general, the flock is reared by following conventional industrial procedures. The mating period commenced in August and September and lasted for 51 days. Lambing took place from the beginning of February to the end of March. At birth, the relevant information about the newborn such as sex, birth type, birth date, birth weight, sir ID and dam ID were recorded. In addition, body weights were recorded at monthly intervals starting from birth until 4 months of age. Body weights for 6, 9 and 12 months of age were also recorded. Lambs were weaned at an average age of 90 days. They were raised separately from older animals until one year of age. During this period, they were not subjected to any form of culling unless they were physically unsound. Animals are kept indoors during winter and receive a ration consisting of wheat and barley straw, alfalfa hay, sugar beet pulp and concentrate. According to the requirement, the food of the ewes was supplemented with concentrates during pregnancy and the nursing period. To protect animals from various diseases, vaccinations were performed twice a year. The sheep were dewormed with drugs and dipped in an anti-parasite bath twice a year. Baluchi sheep selection typically focuses on traits related to growth, reproduction, and adaptability to arid environments. Selection criteria often include body weight, body conformation score, and type of birth. Mating was random for each ram with 15 to 25 ewes. In this flock, about 50% of sires were kept for 2 to 3 mating seasons, and the rest were used just once, and the longevity of ewes was up to 7 lambing (about 8 years of age)^30^.

### Data and pedigree

Birth weight (**BW**) and weaning weight (**WW**) records were extracted from data files. Because lambs were weaned at different ages, weaning weights were adjusted for 90 days of age. Pre-weaning average daily gain (**ADG**) was calculated as total gain divided by the number of days in the period (**WW**-**BW**/90). Errors in the pedigree including repeated animals, animals that were registered as one of their parents and presence the loop in the pedigree were detected and edited with CFC software^31^. Also, animals in the pedigree were re-coded in such a way that the codes for lambs were bigger than their parents’ codes as required for further analyses. The final pedigree included 11,658 animals which were progenies of 258 sirs and 3137 dams (Table 1).

**Table 1:** Pedigree structure of the Baluchi sheep.

No. of generations (including base generation)	17
No. of animals in the pedigree file	11,658
No. of animals with progeny	3395
No. of animals without progeny	8263
No. of non-base animals	10,827
No. of non-base animals with known sire and dam	10,494
No. of non-base animals only with known sire	13
No. of non-base animals only with known dam	320
No. of sire	258
No. of dam	3137
No. of grand sire	210
No. of grand dam	588
No. of great grand sire	181
No. of great grand dam	980

### Statistical analysis

The generalized linear model (GLM) of SAS^32^ was fitted to the data to identify fixed effects of the model. Birth year, lambing age of dam, lamb sex and birth type were significant (*p* < 0.05) for all traits and were subsequently included in the linear mixed models. The WOMBAT program^33^ was used to estimate (co)variance components and genetic parameters. Each trait was analyzed with twelve univariate animal models, including various combinations of additive genetic, dominance genetic, maternal additive genetic and maternal permanent environmental effects (Table 2). As the simplest model, Model 1 included only random animal effects, and Model 12 which was the most complete animal model, included additive genetic, dominance genetic, maternal additive genetic, maternal permanent environmental and covariance between direct and maternal additive genetic effects. The general representation of Model 12 was as follows:

$${\mathbf{y}}\,=\,{\mathbf{Xb}}\,+\,{{\mathbf{Z}}_{\mathbf{1}}}{\mathbf{a}}\,+\,{{\mathbf{Z}}_{\mathbf{2}}}{\mathbf{d}}\,+\,{{\mathbf{Z}}_{\mathbf{3}}}{\mathbf{c}}{\text{ }}+{\text{ }}{{\mathbf{Z}}_{\mathbf{4}}}{\mathbf{m}}{\text{ }}+{\text{ }}{\mathbf{e}},{\text{ cov}}\left( {{\text{a}},{\text{m}}} \right)\, \ne \,0$$

where **y** is the vector of observations. **β** is the vector of fixed effects fitted with design matrix **X**. **Z**_**1**_, **Z**_**2**_, **Z**_**3**_, and **Z**_**4**_ are incidence matrices relating observations to additive genetic, dominance genetic, maternal permanent environmental and maternal additive genetic effects, respectively. The (co)variance matrix for the random effects was as follows:

$$\:Var\:\left[\begin{array}{c}a\\\:d\\\:c\\\:m\\\:e\end{array}\right]=\left[\begin{array}{c}A{{\upsigma\:}}_{\mathbf{a}}^{2}\:\:\:\\\:0\\\:0\\\:{\mathbf{A}{\upsigma\:}}_{\text{a}\text{m}\:\:}\\\:0\end{array}\:\begin{array}{c}0\:\:\\\:D{{\upsigma\:}}_{\text{d}\:\:\:\:}^{2}\\\:0\\\:0\\\:0\end{array}\begin{array}{c}0\\\:0\\\:{\mathbf{I}}_{\text{c}}{{\upsigma\:}}_{\text{c}\:\:\:\:}^{2}\\\:0\\\:0\end{array}\begin{array}{c}{\:\:\:\:\mathbf{A}{\upsigma\:}}_{\text{a}\text{m}\:}\\\:0\\\:0\\\:{\mathbf{A}{\upsigma\:}}_{\text{m}}^{2}\\\:0\end{array}\:\:\:\begin{array}{c}0\\\:0\\\:0\\\:0\\\:{\mathbf{I}}_{\text{e}}{{\upsigma\:}}_{\text{e}}^{2}\end{array}\right]$$

where **a**, **d**, **c**, **m**, and **e** are vectors for additive genetic, dominance genetic, maternal permanent environmental, maternal additive genetic, and residual effects, respectively. $\:{{\upsigma\:}}_{\text{a}}^{2}$,$\:\:{{\upsigma\:}}_{d}^{2}$, $\:{{\upsigma\:}}_{\text{c}}^{2}$, $\:{{\upsigma\:}}_{\text{m}}^{2}$, and $\:{{\upsigma\:}}_{\text{e}}^{2}$ are additive genetic variance, dominance genetic variance, maternal permanent environmental variance, maternal additive genetic variance and residual variance, respectively. **A** and **D** are the additive and dominance numerator relationship matrices, respectively. **I**_**c**_, and **I**_**e**_ are identity matrices of appropriate dimensions. The *nadiv* package^34^ in R software^35^ was used to create matrix **D** and its inverse which was then fitted by the GIN option in WOMBAT^33^. When using the GIN file in WOMBAT^33^, a CODE file and a log determinant value are also required. The CODE file was created using the pedigree file and log determinant was calculated with *nadiv* package^34^. The Akaike’s^36^ information criterion (**AIC**) was computed to rank the models according to their power to fit the data. AIC accounts for both the statistical goodness of fit and the number of parameters that need to be estimated. Let *p* denotes the number of random (co)variance parameters to be estimated, and *Log L* is the maximum likelihood, then the information criterion is defined as **AIC** = -2 *Log L* + 2*p*. The model yielding the smallest **AIC** fits the data best.

**Table 2:** The random (co)variance components fitted in the animal models for growth traits in Baluchi sheep.

Model number	Random effects					
	$\:{\sigma\:}_{a}^{2}$	$\:{\sigma\:}_{d}^{2}$	$\:{\sigma\:}_{m}^{2}$	$\:{\sigma\:}_{c}^{2}$	$\:{\sigma\:}_{a.m}$	$\:{\sigma\:}_{e}^{2}$
1	✓					✓
2	✓	✓				✓
3	✓			✓		✓
4	✓	✓		✓		✓
5	✓		✓			✓
6	✓	✓	✓			✓
7	✓		✓		✓	✓
8	✓	✓	✓		✓	✓
9	✓		✓	✓		✓
10	✓	✓	✓	✓		✓
11	✓		✓	✓	✓	✓
12	✓	✓	✓	✓	✓	✓

$\:{\sigma\:}_{a}^{2}$= additive genetic variance;$\:\:{\sigma\:}_{d}^{2}$= dominance genetic variance; $\:{\sigma\:}_{c}^{2}$=maternal permanent environmental variance;$\:\:{\sigma\:}_{m}^{2}$=maternal genetic variance;$\:\:{\sigma\:}_{a.m}$= direct-maternal additive genetic co-variance; $\:{\sigma\:}_{e}^{2}$= residual variance.

To assess the predictive performance of models, we calculated two indices: (1) the mean squared error of prediction (MSE) as $\:\frac{\sum\:{(yi-\widehat{yi)}}^{2}}{n}$, where *y*_*i*_ and $\:\widehat{{y}_{i}}$ are real and predicted records of animals, and (2) Pearson’s correlation coefficient between real records (y) and predicted values of records ($\:\widehat{y}$) as r($\:y$,$\:\widehat{y}$). The predicted values ​​of the records were obtained from the WOMBAT^33^ outputs.

Correlations between traits were estimated by bi-variate analyses. The models applied in the bi-variate analyses were those selected as best for underlying traits in the univariate analyses. The matrix notation for the bivariate model including **BW** and **WW** was as follows:

$$\begin{aligned} \:\left[ {\begin{array}{*{20}c} {{\mathbf{y}}_{1} } \\ {\:{\mathbf{y}}_{2} } \\ \end{array} } \right] & = \left[ {\begin{array}{*{20}c} {{\mathbf{X}}_{1} } & 0 \\ {\:0} & {{\mathbf{X}}_{2} } \\ \end{array} } \right]\left[ {\begin{array}{*{20}c} {{\mathbf{b}}_{1} } \\ {\:{\mathbf{b}}_{2} } \\ \end{array} } \right] + \left[ {\begin{array}{*{20}c} {{\mathbf{Z}}_{{{\mathbf{a}}1}} } & 0 \\ {\:0} & {{\mathbf{Z}}_{{{\mathbf{a}}2}} } \\ \end{array} } \right]\left[ {\begin{array}{*{20}c} {{\mathbf{a}}_{1} } \\ {\:{\mathbf{a}}_{2} } \\ \end{array} } \right] + \left[ {\begin{array}{*{20}c} {{\mathbf{Z}}_{{{\mathbf{d}}1}} } & 0 \\ {\:0} & {{\mathbf{Z}}_{{{\mathbf{d}}2}} } \\ \end{array} } \right]\left[ {\begin{array}{*{20}c} {{\mathbf{d}}_{1} } \\ {\:{\mathbf{d}}_{2} } \\ \end{array} } \right] \\ & \quad + \left[ {\begin{array}{*{20}c} {{\mathbf{Z}}_{{{\mathbf{c}}1}} } & 0 \\ {\:0} & {{\mathbf{Z}}_{{{\mathbf{c}}2}} } \\ \end{array} } \right]\left[ {\begin{array}{*{20}c} {{\mathbf{c}}_{1} } \\ {\:{\mathbf{c}}_{2} } \\ \end{array} } \right] + \left[ {\begin{array}{*{20}c} {{\mathbf{Z}}_{{{\mathbf{m}}1}} } & 0 \\ {\:0} & 0 \\ \end{array} } \right]\left[ {\begin{array}{*{20}c} {{\mathbf{m}}_{1} } \\ {\:0} \\ \end{array} } \right] + \left[ {\begin{array}{*{20}c} {{\mathbf{e}}_{1} } \\ {\:{\mathbf{e}}_{2} } \\ \end{array} } \right].\: \\ \end{aligned}$$

where **y**_**1**_ and **y**_**2**_ denote traits 1 and 2, respectively.

Estimates of additive breeding values were derived using the best linear unbiased prediction procedure (BLUP) in WOMBAT^33^. The accuracy of estimated additive breeding values for the best model and the best model without dominance effects was calculated as follows:

$$\:{r}_{i}=\sqrt{1-{SE}_{i}^{2}/{\sigma\:}_{a}^{2}}$$

where SE_i_ is the standard error of estimated additive breeding values, derived from the diagonal element of the inverted left-hand side in the mixed model equations and $\:{\sigma\:}_{a}^{2}$ is the additive genetic variance^37^. To determine the significant difference between the accuracy of estimated breeding values of different models, *t* test paired was used.

The effect of the inclusion of dominance effects in the model on additive breeding values was tested by estimating Spearman’s correlation coefficients between additive breeding values obtained by the best model and the best model without dominance effects. It was done for all animals and 10% and 50% of top animals. In addition, change in the ranking of the top 10 and top 50 animals based on their additive breeding values across models was monitored by calculating the number of animals that dropped from the top 10 or top 50 animals after including dominance effects in the model.

For each trait, computing time for the best model and the best model without dominance effects was calculated as the total time used by the CPU to analyze data. The memory used for running these analyses was also measured.

## Results

Table 3 shows the characteristics of the data structure. The mean of **BW**, **WW** and **ADG** were 4.22 kg, 23.51 kg and 212.93 gr, respectively. The variability of body weight at birth (CV = 17.01%) was lower than the variability of weaning weight (CV = 20.88%). Table 4 shows the predictive ability of the models. As shown, models that included dominance effects had lower MSE and higher r($\:y$,$\:\widehat{y}$), i.e., the predictive ability of the models improved by including dominance effects. Estimates of variance components and genetic parameters for **BW**, **WW** and **ADG** are presented in Tables 5, 6 and 7, respectively. For all traits studied, Model 1 which included only additive genetic effects had the least power to fit the data, evidenced by maximum **AIC** values. For all traits studied, models with dominance effects fitted the data substantially better than otherwise identical models, as evidenced by the significant decrease in the **AIC** values. For **BW**, Model 12 which contained additive, dominance and both maternal genetic and permanent environmental effects was the most suitable model. This model had better predictive ability compared to model 11 (model 12 without dominance genetic effects) as it provided lower MSE and higher r($\:y$,$\:\widehat{y}$). For **WW** and **ADG**, it was model 4 that fitted the data best. It included additive, dominance and maternal permanent environmental effects and showed better predictive ability compared to model 3 (model 4 without dominance genetic effects) according to estimates of MSE and r($\:y$,$\:\widehat{y}$).

**Table 3:** Characteristics of the data structure in Baluchi sheep.

	BW (kg)	WW (kg)	ADG (g day^−1^)
No. of records	7339	7300	6451
No. of sires with progeny	180	209	176
No. of sires with progeny and record	93	100	89
Average number of progeny per sire	40.77	34.92	36.65
No. of dams with progeny	2141	2227	2047
No. of dams with progeny and record	1540	1685	1452
Average number of progeny per dam	3.42	3.27	3.15
Mean	4.22	23.51	212.93
Min.	2.10	10.00	50
Max.	6.50	37.81	359
SD	0.718	4.908	52.01
CV (%)	17.01	20.88	24.42

*BW=* birth weight, *WW*=weaning weight, *ADG=* average daily gain from birth to weaning, *SD=* standard deviation, *CV=* coefficient of variation.

**Table 4:** Predictive ability of the best model and the best model without dominance effects.

Trait	*r*($\:y$,$\:\widehat{y}$)		MSE	
	Best model	Best model without Dominance effects	Best model	Best model without Dominance effects
**BW**	0.95	0.70	0.29	0.65
**WW**	0.86	0.70	8.84	13.43
**ADG**	0.88	0.73	880.98	1470.54

*BW=* birth weight, *WW=* weaning weight, *ADG=* average daily gain from birth to weaning, *MSE=* Mean squared error of prediction. r($\:y$,$\:\widehat{y}$) = Pearson’s correlation coefficient between the real and predicted values of records.

**Table 5:** Estimates of variance components and genetic parameters for birth weight (the best model is shown in bold).

Model	$\:{\sigma\:}_{a}^{2}$	$\:{\sigma\:}_{d}^{2}$	$\:{\sigma\:}_{c}^{2}$	$\:{\sigma\:}_{m}^{2}$	$\:{\sigma\:}_{a.m}$	$\:{\sigma\:}_{e}^{2}$	$\:{\sigma\:}_{p}^{2}$	$\:{h}_{a}^{2}$	$\:{h}_{d}^{2}$	$\:{h}_{c}^{2}$	$\:{h}_{m}^{2}$	e^2^	*r*_a, m_	AIC
1	0.196					0.348	0.544	0.36 ± 0.03				0.64 ± 0.03		1068.69
2	0.194	0.191				0.162	0.548	0.35 ± 0.03	0.35 ± 0.06			0.30 ± 0.07		1054.69
3	0.052		0.112			0.347	0.511	0.10 ± 0.02				0.68 ± 0.02		911.59
4	0.050	0.147	0.111			0.205	0.514	0.10 ± 0.03	0.29 ± 0.06			0.39 ± 0.06		901.60
5	0.023			0.155		0.368	0.547	0.04 ± 0.01		0.22 ± 0.02	0.28 ± 0.02	0.67 ± 0.02		914.01
6	0.022	0.159		0.153		0.214	0.550	0.04 ± 0.01	0.29 ± 0.06	0.22 ± 0.02	0.28 ± 0.02	0.39 ± 0.06		902.16
7	0.021			0.138	0.035	0.370	0.549	0.04 ± 0.01			0.25 ± 0.02	0.67 ± 0.02	0.35 ± 0.19	913.63
8	0.020	0.160		0.136	0.019	0.215	0.551	0.04 ± 0.02	0.29 ± 0.06		0.24 ± 0.02	0.39 ± 0.05	0.37 ± 0.19	901.70
9	0.027		0.068	0.061		0.359	0.517	0.05 ± 0.02		0.13 ± 0.02	0.11 ± 0.02	0.69 ± 0.02		894.67
10	0.026	0.151	0.066	0.062		0.213	0.520	0.05 ± 0.01	0.30 ± 0.06	0.13 ± 0.02	0.12 ± 0.02	0.41 ± 0.06		883.96
11	0.024		0.067	0.048	0.017	0.361	0.519	0.05 ± 0.01		0.13 ± 0.02	0.09 ± 0.02	0.70 ± 0.01	0.49 ± 0.22	893.42
12	**0.023**	**0.152**	**0.065**	**0.049**	**0.017**	**0.214**	**0.514**	**0.05 ± 0.01**	**0.29 ± 0.06**	**0.13 ± 0.02**	**0.09 ± 0.02**	**0.41 ± 0.03**	**0.50 ± 0.22**	**882.64**

$\:{\sigma\:}_{a}^{2}$= additive genetic variance;$\:\:{\sigma\:}_{d}^{2}$= dominance genetic variance;$\:\:{\sigma\:}_{c}^{2}$= maternal permanent environmental variance;$\:\:{\sigma\:}_{m}^{2}$= maternal genetic variance; $\:{\sigma\:}_{a.m}$= direct-maternal additive genetic covariance; $\:{\sigma\:}_{e}^{2}$= residual variance; $\:{\sigma\:}_{p}^{2}$= phenotypic variance;$\:\:{h}_{a}^{2}$= additive heritability; $\:{h}_{d}^{2}$= dominance heritability; $\:\:{h}_{c}^{2}$= maternal permanent environmental effect, $\:{h}_{m}^{2}$= maternal heritability; **e**^2^: ratio of residual variance to phenotypic variance; *r*_*a, m*_= direct-maternal additive genetic correlation; AIC= Akaike’s information criterion.

**Table 6:** Estimates of variance components and genetic parameters for weaning weight (the best model is shown in bold).

Model	$\:{\sigma\:}_{a}^{2}$	$\:{\sigma\:}_{d}^{2}$	$\:{\sigma\:}_{c}^{2}$	$\:{\sigma\:}_{m}^{2}$	$\:{\sigma\:}_{a.m}$	$\:{\sigma\:}_{e}^{2}$	$\:{\sigma\:}_{p}^{2}$	$\:{h}_{a}^{2}$	$\:{h}_{d}^{2}$	$\:{h}_{c}^{2}$	$\:{h}_{m}^{2}$	e^2^	*r*_a, m_	AIC
1	4.551					16.750	21.302	0.21 ± 0.03				0.78 ± 0.02		14637.48
2	4.550	2.697				14.115	21.346	0.21 ± 0.02	0.13 ± 0.07			0.66 ± 0.07		14636.69
3	1.632		3.032			16.048	20.713	0.08 ± 0.03		0.15 ± 0.01		0.78 ± 0.02		14559.81
4	**1.629**	**3.114**	**3.045**			**13.005**	**20.794**	**0.08 ± 0.02**	**0.15 ± 0.07**	**0.15 ± 0.01**		**0.62 ± 0.07**		**14558.33**
5	1.252			3.277		16.847	21.377	0.06 ± 0.02			0.15 ± 0.02	0.79 ± 0.02		14585.06
6	1.249	3.054		3.296		13.860	21.640	0.06 ± 0.02	0.14 ± 0.06		0.15 ± 0.02	0.65 ± 0.07		14583.73
7	1.240			3.244	0.036	16.855	21.378	0.06 ± 0.02			0.15 ± 0.02	0.79 ± 0.02	0.02 ± 0.17	14586.06
8	1.235	3.055		3.253	0.047	13.870	21.641	0.06 ± 0.02	0.14 ± 0.06		0.15 ± 0.02	0.65 ± 0.07	0.02 ± 0.19	14584.73
9	1.431		2.666	0.478		16.153	20.729	0.07 ± 0.02		0.13 ± 0.01	0.02 ± 0.01	0.78 ± 0.02		14559.49
10	1.428	3.117	2.678	0.479		13.106	20.811	0.07 ± 0.02	0.15 ± 0.06	0.13 ± 0.01	0.02 ± 0.01	0.63 ± 0.07		14558.01
11	1.315		2.648	0.336	0.208	16.227	20.736	0.06 ± 0.02		0.13 ± 0.01	0.02 ± 0.01	0.78 ± 0.02	0.31 ± 0.27	14560.29
12	1.355	3.134	2.660	0.327	0.221	13.168	20.818	0.06 ± 0.02	0.15 ± 0.06	0.13 ± 0.01	0.02 ± 0.01	0.64 ± 0.07	0.34 ± 0.40	14558.79

$\:{\sigma\:}_{a}^{2}$= additive genetic variance;$\:\:{\sigma\:}_{d}^{2}$= dominance genetic variance;$\:\:{\sigma\:}_{c}^{2}$= maternal permanent environmental variance;$\:\:{\sigma\:}_{m}^{2}$= maternal genetic variance; $\:{\sigma\:}_{a.m}$= direct-maternal additive genetic covariance; $\:{\sigma\:}_{e}^{2}$= residual variance; $\:{\sigma\:}_{p}^{2}$= phenotypic variance;$\:\:{h}_{a}^{2}$= additive heritability; $\:{h}_{d}^{2}$= dominance heritability; $\:\:{h}_{c}^{2}$= maternal permanent environmental effect, $\:{h}_{m}^{2}$= maternal heritability; **e**^2^: ratio of residual variance to phenotypic variance; *r*_*a, m*_= direct-maternal additive genetic correlation; AIC= Akaike’s information criterion.

**Table 7:** Estimates of variance components and genetic parameters for average daily gain (the best model is shown in bold).

Model	$\:{\sigma\:}_{a}^{2}$	$\:{\sigma\:}_{d}^{2}$	$\:{\sigma\:}_{c}^{2}$	$\:{\sigma\:}_{m}^{2}$	$\:{\sigma\:}_{a.m}$	$\:{\sigma\:}_{e}^{2}$	$\:{\sigma\:}_{p}^{2}$	$\:{h}_{a}^{2}$	$\:{h}_{d}^{2}$	$\:{h}_{c}^{2}$	$\:{h}_{m}^{2}$	e^2^	*r*_a, m_	AIC
1	453.571					1871.31	3324.80	0.19 ± 0.03				0.81 ± 0.02		28034.95
2	450.776	405.77				1477.34	2333.23	0.19 ± 0.03	0.17 ± 0.07			0.63 ± 0.07		28033.12
3	171.902		277.506			1816.85	2366.26	0.08 ± 0.03		0.12 ± 0.01		0.80 ± 0.02		27996.11
4	**168.513**	**460.623**	**279.947**			**1368.25**	**2277.34**	**0.07 ± 0.02**	**0.20 ± 0.07**	**0.12 ± 0.01**		**0.60 ± 0.07**		**27983.08**
5	142.859			272.98		1902.07	2317.91	0.06 ± 0.02			0.12 ± 0.02	0.82 ± 0.02		28008.67
6	138.053	451.382		277.043		1462.83	2329.31	0.06 ± 0.02	0.19 ± 0.07		0.12 ± 0.02	0.63 ± 0.07		28006.10
7	139.954			261.990	12.421	1904.76	2318.13	0.06 ± 0.02			0.11 ± 0.02	0.82 ± 0.02	0.06 ± 0.21	28009.64
8	134.623	451.539		264.600	13.487	1456.39	2329.64	0.06 ± 0.02	0.19 ± 0.07		0.11 ± 0.02	0.62 ± 0.07	0.07 ± 0.23	28007.08
9	161.272		264.03	18.672		1822.19	2266.17	0.07 ± 0.02		0.12 ± 0.02	0.01 ± 0.02	0.80 ± 0.02		27986.73
10	157.192	461.381	266.395	18.890		1373.28	2277.39	0.07 ± 0.02	0.20 ± 0.07	0.12 ± 0.02	0.01 ± 0.02	0.61 ± 0.07		27983.85
11	156.485		262.610	14.804	7.352	1825.14	2266.40	0.07 ± 0.02		0.12 ± 0.01	0.01 ± 0.02	0.80 ± 0.02	0.15 ± 0.69	27987.71
12	150.757	462.83	264.429	13.515	10.135	1375.85	2277.52	0.07 ± 0.02	0.20 ± 0.07	0.12 ± 0.01	0.01 ± 0.02	0.60 ± 0.07	0.22 ± 0.77	27984.80

$\:{\sigma\:}_{a}^{2}$= additive genetic variance;$\:\:{\sigma\:}_{d}^{2}$= dominance genetic variance;$\:\:{\sigma\:}_{c}^{2}$= maternal permanent environmental variance;$\:\:{\sigma\:}_{m}^{2}$= maternal genetic variance; $\:{\sigma\:}_{a.m}$=direct-maternal additive genetic covariance; $\:{\sigma\:}_{e}^{2}$= residual variance; $\:{\sigma\:}_{p}^{2}$= phenotypic variance;$\:\:{h}_{a}^{2}$= additive heritability; $\:{h}_{d}^{2}$= dominance heritability; $\:\:{h}_{c}^{2}$= maternal permanent environmental effect, $\:{h}_{m}^{2}$= maternal heritability; **e**^2^: ratio of residual variance to phenotypic variance; *r*_*a, m*_= direct-maternal additive genetic correlation; AIC= Akaike’s information criterion.

By including dominance effects in the model, additive genetic variance did not change, but residual variance decreased significantly by 41%, 21% and 25% for **BW**, **WW** and **ADG**, respectively, which indicated that dominance effects distangelled from residual variance. For **BW**, **WW** and **ADG**, dominance genetic variance was 6.61, 1.91, and 2.73 times greater than additive genetic variance and contributed 87%, 65% and 73% to the total genetic variance of **BW**, **WW** and **ADG**, respectively.

Estimates of dominance heritability ($\:{\varvec{h}}_{\varvec{d}}^{2}$), were 0.29 ± 0.06, 0.15 ± 0.07 and 0.20 ± 0.07 for **BW**, **WW** and **ADG**, respectively. Additive heritability ($\:{\varvec{h}}_{\varvec{a}}^{2}$), was 0.05 ± 0.01 for **BW**, 0.08 ± 0.02 for **WW** and 0.07 ± 0.02 for **ADG**, respectively. Maternal permanent environmental effects ($\:{\varvec{h}}_{\varvec{c}}^{2}$) were 0.13 ± 0.02, 0.15 ± 0.01 and 0.12 ± 0.01 for **BW**, **WW** and **ADG**, respectively. For **BW** a maternal genetic component was also significant which contributed 9% to the phenotypic variance.

The accuracy of additive breeding values for traits studied estimated by the best model and the best model without dominance effects are listed in Table 8. As shown, models with dominance effects provided additive breeding values with higher accuracy. For **BW**, **WW** and **ADG**, by including dominance effects in the model, the accuracy of additive breeding values increased by 8%, 8% and 11%, respectively.

**Table 8:** Accuracy of breeding values estimated by the best model and the best model without dominance effects for studied traits.

Model	BW	WW	ADG
Best model	0.571^a^	0.634^a^	0.496^a^
Best model without dominance effects	0.527^b^	0.586^b^	0.447^b^

*BW=* birth weight, *WW=* weaning weight, *ADG=* average daily gain from birth to weaning.

Values within columns that do not have a common superscript are significantly different (*p*<0.05).

Correlation between additive breeding values obtained from the best model and the best model without dominance effects for all animals, top 50% ranked animals and top 10% ranked animals are shown in Table 9. As shown correlations were close to unity for all traits studied, indicating negligible changes in the additive breeding values after the inclusion of dominance effects in the model. On average, 9.7 animals out of the top 10 and 49.3 animals out of the top 50 were common when additive breeding values of lambs were estimated using the best model and the best model without dominance effects (Table 10).

**Table 9:** Correlations between breeding values predicted by the best model and the best model without dominance effects.

Parameters	BW	WW	ADG
All animals	1.00	1.00	1.00
Top 50% ranked	0.998	1.00	0.998
Top 10% ranked	0.996	0.997	0.999

*BW=* birth weight, *WW=* weaning weight, *ADG=* average daily gain from birth to weaning.

**Table 10:** The number of animals that were common between the best model and the best model without dominance effects based on their additive breeding values.

Trait	Top 10	Top 50
BW	9	48
WW	10	50
ADG	10	50
Overall mean	9.66	49.33

*BW=* birth weight, *WW=* weaning weight, *ADG=* average daily gain from birth to weaning.

Correlations between traits are listed in Table 11. Additive genetic correlation (***r***_***a***_), maternal permanent environmental correlation (***r***_***c***_) and phenotypic correlation (***r***_***p***_) were all positive, ranging from 0.21 ± 0.09 to 0.87 ± 0.04. Dominance genetic correlation (***r***_***d***_) between **WW** and **ADG** was positively high (0.99 ± 0.45) and between other pairs of traits was negative.

**Table 11:** Correlations between studied traits.

Trait 1	Trait 2	*r*_a_	*r*_d_	*r*_c_	*r*_*p*_
BW	WW	0.80 ± 0.05	− 0.73 ± 0.54	0.40 ± 0.07	0.39 ± 0.01
	ADG	0.73 ± 0.07	− 0.62 ± 0.54	0.21 ± 0.09	0.27 ± 0.01
WW	ADG	0.87 ± 0.04	0.99 ± 0.45	0.80 ± 0.04	0.78 ± 0.01

*r*_*a*_= additive genetic correlation; *r*_*d*_= dominance genetic correlation; *r*_*c*_= maternal permanent environmental correlation; *r*_*p*_= phenotypic correlation. BW = birth weight; WW = weaning weight; ADG = average daily gain from birth to weaning.

**Fig. 1:**
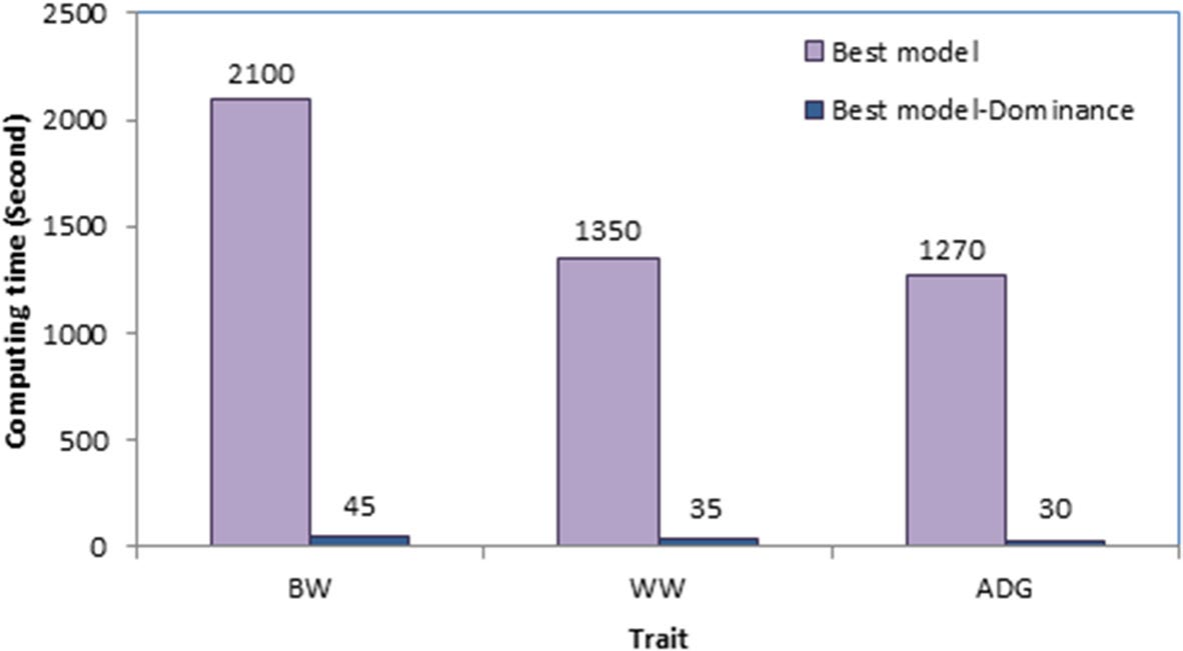
Computing time for analyses with and without dominance effects.

**Fig. 2:**
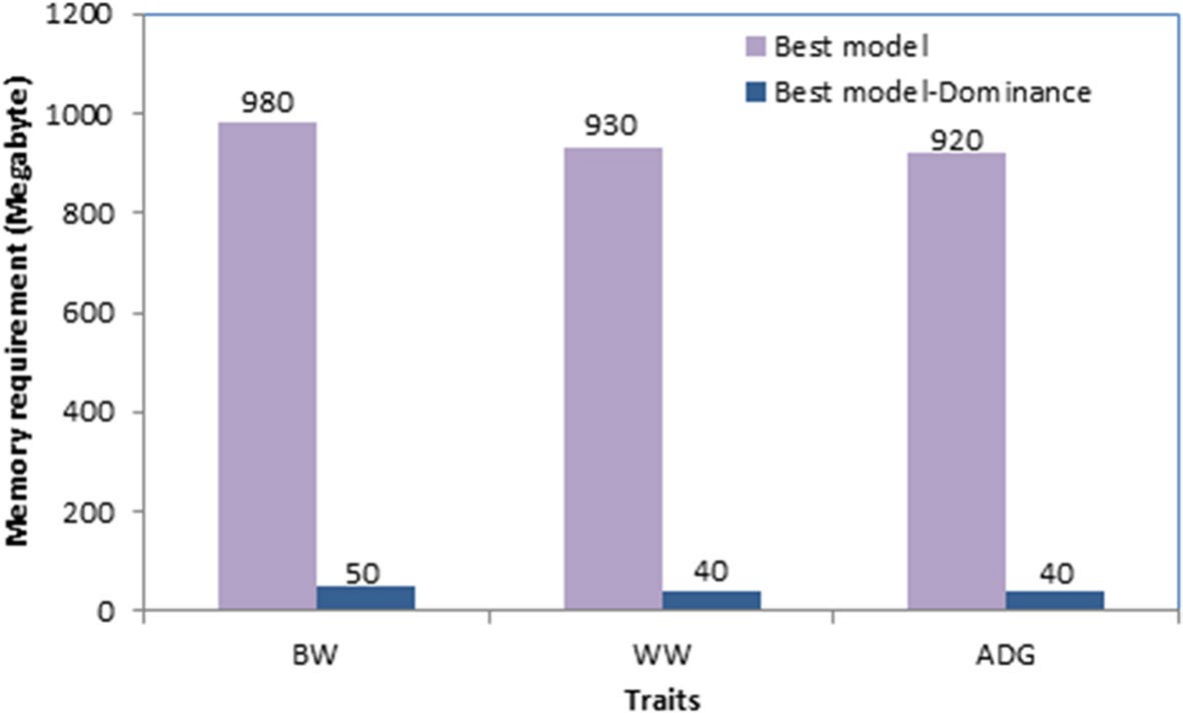
Memory requirement for analyses with and without dominance effects.

Computing time for the best models and the best models without dominance effects is shown in Fig. 1. For the best models which included dominance effects, analyses took 2100, 1350 and 1270 s for **BW**, **WW** and **ADG**, respectively; significantly higher than those observed for best models without dominance effects (45, 35 and 30 s for **BW**, **WW** and **ADG**, respectively). In addition, for all traits, running the best model consumed ≈ 1 gigabyte of memory, much more than analyses without dominance effects (≈ 50 megabytes) (Fig. 2). Moreover, constructing dominance genetic relationship matrix and its inverse with the *nadiv* package^34^ needed ≈ 30 gigabytes of memory (not shown).

## Discussion

Despite decades of theoretical and experimental efforts, the quantification of non-additive genetic variation in livestock populations such as sheep remains challenging, leading to neglecting these effects from the genetic evaluation models. One of the reasons for neglecting dominance effects from the genetic evaluation process is presented in Fig. 1. As shown, including dominance effects in the model increased computing time between 42 and 47 times, depending on the trait. In addition, it needed a huge amount of memory (≈ 20 times). Therefore, longer computing time and higher memory requirements can be the “Achilles Heel” of such analyses. Our data was not a big dataset, nonetheless, for computing inverse of the relationship matricx for dominance effects from our pedigree (including 11658 individuals), *nadiv* package^34^ consumed 30 Gigabytes of RAM. Obviously, in dealing with big data, the analyses may take several hours and need a huge amount of memory. Therefore, due to high computational demand, analysis of big datasets may not be possible with conventional PCs. However, current results showed that although presenting dominance effects to the model significantly increased the computing burden, it increased the likelihood and predictive ability of the fitting model as well as the accuracy of the additive breeding values. Therefore, an additive + dominance model can be superior to a purely additive model in better unraveling the genetic variance components, leading to a more accurate and precise estimation of genetic parameters. Jasouri et al.^25^ and Liu et al.^16^ in chicken and Sadeghi et al.^24^ and Sadeghi et al.^15^ in goat reported improvement in general properties of the model by including dominance effects which is in line with our findings.

Our finding showed that dominance effects were part of residual variance and did not separate from additive genetic variance. Therefore, excluding dominance effects did not cause inflated additive genetic variance. Using genomic data, Moghaddar and van der Werf^12^ estimated additive and dominance genetic variances for body weight and body composition traits in Merino sheep and reported a notably lower residual variance in models containing the dominance effect. Heidaritabar et al.^38^ reported that dominance and epistasis effects were important for egg production traits in layers and when these effects were ignored from the genomic evaluation models, they were accumulated in residual variance. In Adani goat, Sadeghi et al.^24^ worked on body weight traits and reported that by including dominance effects, residual variance decreased in a range between 28.2% (weaning weight) to 59.2% (birth weight). Also, Sadeghi et al.^15^ reported a decrease in residual variance between 20.6% (ADG from 6 to 9 months of age) to 50.21% (ADG from weaning to 3 months of age) in Adani goat. A slight non-significant change in the estimation of additive genetic variance following including dominance effects in the model indicated some confounding between random effects. The confounding between additive and non-additive genetic effects based on pedigree has been reported in the literature^39,40^. Nishio and Satoh^41^ similarly reported a slight change in the estimation of additive genetic variance following including dominance effects in the model. Data size determines the magnitude of confounding between additive and non-additive genetic effects. The smaller the data size, the more will be the confounding between additive and non-additive genetic effects^12^.

There is a general scarcity regarding dominance heritability ($\:{\varvec{h}}_{\varvec{d}}^{2}$) in the literatures for body weight traits in sheep. Using genomic information, some authors tried to estimate the relative contribution of dominance effects to economic traits of sheep. For example Moghaddar and van der Werf^12^ estimated dominance heritability ($\:{\varvec{h}}_{\varvec{d}}^{2}$) for **BW** and **WW** of Merino sheep as 0.07 and 0.11. In Alpine Merino sheep, a large component of phenotypic variation for fleece extension rate ($\:{\varvec{h}}_{\varvec{d}}^{2}$=0.73), red blood cell count ($\:{\varvec{h}}_{\varvec{d}}^{2}$=0.28), and hematocrit ($\:{\varvec{h}}_{\varvec{d}}^{2}$=0.25), was explained by dominance effects^42^. In other livestock species, dominance heritability is available for growth traits estimated using conventional animal models. For example, Sadeghi et al.^24^ worked on Adani goats and reported $\:{\varvec{h}}_{\varvec{d}}^{2}$ for body weight at birth, weaning, six, nine and twelve months of age as 0.15, 0.17, 0.11, 0.19 and 0.25, respectively, smaller than estimates of additive heritability ($\:{\varvec{h}}_{\varvec{a}}^{2}$) (0.35, 0.18, 0.36, 0.28 and 0.28, respectively). In addition, Sadeghi et al.^15^ estimated $\:{\varvec{h}}_{\varvec{d}}^{2}$ for pre- and post-weaning average daily gain of Adani goat as 0.15 and 0.11, respectively. Heidaritabar et al.^38^ reported $\:{\varvec{h}}_{\varvec{d}}^{2}$ for egg production, average egg weight, albumin height, egg color, yolk weight and age at sexual maturity for brown layers as 0.14, 0.22, 0.22, 0.20, 0.13 and 0.13, respectively. Jasouri et al.^25^ who worked on Iranian native fowl, reported $\:{\varvec{h}}_{\varvec{d}}^{2}$ for body weight at birth, eight weeks and twelve weeks of age as 0.06, 0.08 and 0.01, respectively. In addition, $\:{\varvec{h}}_{\varvec{d}}^{2}$ was 0.06, 0.06 and 0.08 for the age at sexual maturity, average egg weight and number of eggs, respectively. Li et al.^43^ reported that the dominant variance of broiler feed-related traits accounted for 29.5–58.4% of the genetic variance. These studies together with our findings show that dominance effects are an important component of phenotypic values.

Current estimates of $\:{\varvec{h}}_{\varvec{a}}^{2}$ are within the range of other reports in Iranian sheep breeds^29,44–49^. The heritability of a trait corresponds to the fraction of the selection differential that can cause a genetic change in the offspring generation. The heritability thus acts as a filter that determines how efficiently a population can respond to phenotypic selection^50^. Our estimates of $\:{\varvec{h}}_{\varvec{a}}^{2}$ for traits studied are below 0.1 which indicates that a limited response could be expected following the selection on these traits. Strong directional selection is predicted to erode additive genetic variance and, subsequently, decrease the heritability of a trait. As a consequence, the response to selection will be reduced^51^.

Estimates of dominance heritability were higher than additive heritability which did not agree with Moghaddar and van der Werf^12^, Jasouri et al.^25^, Sadeghi et al.^24^ and Sadeghi et al.^15^. This may be explained, to some extent, by the effect of the genetic structure of the populations and size of the data used in different studies. Another explanation for this finding is the Fisher’s^52^ hypotheses associated with his theory of dominance which predict that traits closely associated with fitness should have a significant dominance variance component, both due to the erosion of the additive component of variance and the evolution of directional dominance. Therefore, in addition to eroding additive variance, selection is also expected to act directly on genetic dominance, resulting in a further relative increase of dominance variance to total genetic variance^13^. Growth traits may have been correlated with fitness in the ancestral populations from which the contemporary Baluchi sheep has been drawn.

The results show that dominance variance was higher in lowly heritable traits. The heritability of **BW** (0.05) was lower than **WW** (0.08), but dominance heritability was higher for **BW** (0.29) compared to **WW** (0.15). Comparing **WW** with **ADG** and/or **BW** with **ADG**, a similar result was observed. Similarly, Moghaddar and van der Werf^12^ and Sadeghi et al.^24^ reported higher $\:{\varvec{h}}_{\varvec{d}}^{2}$ for traits with lower $\:{\varvec{h}}_{\varvec{a}}^{2}$, though there were expectations in both studies. However, more research is needed to have a clear-cut verdict about this finding. It is notable that data size plays a significant role in estimating dominance variance and, consequently, dominance heritability. Small data size has been reported as a potential reason for observing almost no dominance effect for body composition traits in sheep^12^.

Spearman’s correlation between the additive breeding values obtained from the best model and the best model without the dominance effects was high and close to unity indicating little change in additive breeding values after presenting dominance effects to the model. A correlation close to 1.00 means that the ranking of animals may not change across models. The later result is supported by information in Table 10 which shows that out of 10 and 50 top animals, nearly all of them remained in their groups after presenting dominance effects to the model. In other words, the ranking of top animals did not change across models. However, an increase in the accuracy of additive breeding values after including dominance effect in the genetic evaluation model has been frequently reported by Toro and Varona^53^, Duenk et al.^23^, Sadeghi et al.^24^ and Sadeghi et al.^15^ which are in agreement with our findings. It means that although the inclusion of dominance effects in the model, may not change the ranking of top animals, it increases the accuracy of estimated additive breeding values which means the accurate prediction of selection response. On the other hand, there are reports indicating a small improvement (2.3%) in the accuracy of genomic breeding values for body weight and body composition traits in Merino sheep as a result of accounting for the dominance effect^12^. They stated that it was because of the small variance component of the dominance effect in the studied traits, i.e., whatever the contribution of dominance effects is higher, the greater will be the increase in the accuracy of additive breeding values after the inclusion of dominance effects. Accordingly, Moghadar et al.^12^ reported that using additive + dominance models improved the accuracy of genomic evaluation for traits with higher dominance variation.

Positive additive genetic correlation between studied traits allows for improving all traits simultaneously. In agreement with our findings, some authors including Mokhtari et al.^54^ Eskandarinasab et al.^44^ and Singh et al.^10^ reported the positive additive genetic correlation between the growth traits of different breeds of sheep. Additive genetic correlation is the heritable relationship between traits. Although, from a breeding perspective, a positive additive genetic correlation between growth traits is preferred, a negative genetic correlation may also be desirable. For example, negative genetic correlation between two traits may limit the erosion of genetic variance of both traits by inducing a response of one trait to selection pressures on the other (correlational selection)^55^. In this study, for the first time, dominance genetic correlations between growth traits in sheep are estimated. Dominance genetic correlations between **BW** and **WW** and between **BW** and **ADG** were negative. Positive ***r***_***a***_ between **BW** and **WW** means that the value of **BW** in parents is correlated to the value of **WW** in offspring. Regarding dominance genetic correlation, regardless of whether the ***r***_***d***_ is positive or negative, the value of **BW** in the parent does not correlate to the **WW** value in offspring. While the additive genetic correlation would accelerate the response (if both traits were under the same direction of selection), the dominance correlation is not heritable, so it does not contribute to the response to selection (and hence doesn’t accelerate the response). The SE of dominance genetic correlations is higher than additive genetic correlations. In animal models, SE is an indicator of data size, data structure, and deep and quality of pedigree used^49^. Gerstmayr^56^ reported that the estimation of genetic correlations was more sensitive to data size and data structure than the estimation of heritabilities and that a larger sample is required to estimate a genetic correlation with the same accuracy as for heritability. In addition, the frequency of full-sib families in the population is also important because dominance effects contribute to the (co)variances between full-sib relatives. As a result, compared with additive genetic parameters, to have accurate estimates of dominance genetic parameters, a relatively bigger data size including a decent frequency of full-sib families is needed.

In conclusion, dominance effects significantly contributed to the phenotypic variation of body weight and average daily gain in Baluchi lambs. Accounting for dominance effects improved the likelihood and predictive ability of the model. A direct consequence would be more precise and accurate estimates of variance components and additive breeding value. In addition, an increase in the accuracy of additive breeding values was observed after presenting dominance effects to the model. However, accounting for dominance effects significantly increased the computing burden. Correlation between breeding values estimated by models with and without dominance effects was close to unity, indicating a little chance for re-ranking of top animals after inclusion of dominance effects in the model. While additive genetic correlations between traits were positively high, dominance genetic correlations were negative in 2 of 3 cases. Since the inclusion of dominance effects improved the general properties of the model and increased the accuracy of additive breeding values, a model including dominance effects would have an advantage over a purely additive model in unraveling the genetic variance components and prediction of breeding values.

## Data Availability

The data that support the findings of this study have been deposited in the National Animal Breeding Center and Promotion of Animal Products (https://www.abc.org.ir/).

## Abbreviations

BW: Birth weight
WW: Weaning weight
ADG: Average daily gain
AIC: Akaike’s information criterion
REML: Restricted maximum likelihood
GLM: Generalized linear model
BLUP: Best linear unbiased prediction
MSE: Mean squared error of prediction
